# Gender Differences in Psychosocial, Religious, and Spiritual Aspects in Coping: A Cross-Sectional Study with Cancer Patients

**DOI:** 10.1089/whr.2021.0012

**Published:** 2021-10-04

**Authors:** Anahita Rassoulian, Alexander Gaiger, Henriette Loeffler-Stastka

**Affiliations:** ^1^ Department of Psychoanalysis and Psychotherapy, Medical University of Vienna, Vienna, Austria.; ^2^ Division of Hematology and Hemostaseology, Department of Internal Medicine, Medical University of Vienna, Vienna, Austria.

**Keywords:** cancer, gender differences, religion, religious coping, psychosocial, spirituality

## Abstract

***Background:*** There is a growing awareness of religiosity and/or spirituality (R/S) as a possible resource in coping with cancer. Gender differences in religious coping have not yet been thoroughly examined. This study aimed to analyze differences in religious coping between men and women with cancer and compare the impact of R/S on anxiety and depression symptoms.

***Methods:*** This cross-sectional study was conducted at the Divisions of Hematology and Oncology of the Medical University of Vienna. In total, 352 patients with a cancer diagnosis, who regarded themselves as religious and/or spiritual, were interviewed at Vienna's university hospital with standardized questionnaires. To answer our research questions, we used the Hospital Anxiety and Depression Scale (HADS), the Benefit Through Spirituality/Religiosity (Benefit) questionnaire, and collected demographic characteristics.

***Results:*** Of 689 cancer patients, 51% (352) regard themselves as religious and/or spiritual. Women with cancer tend toward R/S more significantly (57%) than men (45%). In patients with an R/S belief, women scored higher in almost all items of the Benefit questionnaire and showed higher prevalence of anxiety (*p* < 0.001) and depression than men. Regarding the socioeconomic characteristics, more women were widowed, and had significantly lower income than men.

***Conclusions:*** The results show a significant gender gap concerning the importance of R/S for cancer patients and the effect on psychological well-being.

Women in this study were more religious/spiritual than men and scored higher on anxiety and depression. We support the notion that the gender perspective is essential and can contribute to better patient care in identifying gender-specific concerns.

## Introduction

The diagnosis and treatment of cancer are often combined with existential challenges. Studies indicate that religiosity and/or spirituality (R/S) play an important and supportive role in patients' cancer experience.^1–3^ Higher levels of religious and spiritual engagement are associated with a better quality of life and well-being.^4,5^ For many cancer patients R/S can offer a source of strength, hope, and comfort^6,7^ and contribute to greater psychological well-being.^8,9^ The use of R/S as one of the coping strategies in dealing with a life-threatening disease can also be influenced by cultural and sociodemographic aspects.^10,11^ Studies describe an association between age, gender, education, and income and higher levels of R/S.^12–14^

Cancer patients often suffer from psychological distress. Anxiety and depression are found to be frequent reactions among cancer patients.^15–17^ According to Mehnert et al.,^18^ who examined the 4-week prevalence of mental health disorders in 5889 cancer patients, the anxiety disorders were the most prevalent, and 1 in 3 cancer patients had a need for psycho-oncological interventions. It is also well documented that women with cancer suffer more with anxiety and depression and have a lower quality of life.^19,20^ In contrast, men show different symptoms of psychological distress and find it more difficult to express emotions and need for help.^21,22^

R/S can be a buffer against psychological distress. Studies demonstrated a significant relationship between R/S and lower anxiety and depression levels in cancer patients^23,24^ and a better acceptance of illness.^25^ Performing R/S practices such as praying, yoga, meditation, or others contributes to better well-being and physical health.^26^ Studies have demonstrated reduced feelings of pain, fatigue, and anxiety in people with R/S practices.^27–29^ Research has highlighted the crucial role of religious coping in men and women with cancer. However, little is known about the gender differences in religious coping with cancer.

It is generally known that in a Christian society, women tend more toward R/S and also more toward religious/spiritual practices than men.^14,30,31^ But does this mean that women subsequently deal with their disease better than men?

The aim of this study was to analyze whether R/S can be a supportive coping mechanism in the cancer journey and whether there are differences in the adherence to and practice of R/S between men and women.

Second, we wanted to examine the effects of the interaction between R/S and psychological distress, such as anxiety and depression.

## Methods

The cross-sectional study was conducted at the Department of Medicine I, Divisions of Hematology and Oncology of the Medical University of Vienna and included 689 patients. All patients were recruited from April 2013 to June 2014. The data presented here are part of a broader research project, including 689 patients, to examine the differences between patients with and without an R/S in coping with cancer.

For this presented study, we only included patients with an R/S (352) and focused on gender differences. Patients between 18 and 90 years with a cancer diagnosis who understood German and were willing to contribute were included with no regard to gender, age, origin, religious affiliation, tumor entities, or disease stages. Patients who were unable to answer the questionnaires due to medical conditions at the time of the survey were excluded from the study. Patients were interviewed by members of the psycho-oncology team during their visit at the clinic of hematology and oncology of the Medical University of Vienna and were free to decline attendance. The sample size was given by patients answering our questionnaires during the study period. The study was performed in accordance with the Declaration of Helsinki and was approved by the ethics committee of the medical university of Vienna, Austria (EK-number: 473/2006).

### Instruments

For assessing anxiety and depression in our participants, we used the valid and self-administered Hospital Anxiety and Depression Scale (HADS) questionnaire, which has 14 items, 7 of which are for anxiety and 7 for depression. The answers consist of scores between 0 and 3. For the analysis, all items are added, and the total score makes a classification (0–7 = normal, 8–10 = moderate, 11–21 = severe).^32^ Both anxiety and depression have seven questions each, allowing for a maximum score of 21 points. This classification only serves to point out a higher amount of anxiety or depression and does not allow for a final diagnosis. The HADS has a good internal reliability, with a Cronbach's alpha score for both subscales between 0.82 and 0.90.

In addition, we used the self-administered Benefit Through Spirituality/Religiosity (Benefit) questionnaire developed by Büssing, at the Medical University of Witten Herdecke, Germany. It was designed to analyze whether patients perceive their R/S as having a positive effect on various aspects of their life, including experiences with serious illness.

The six items of the Benefit questionnaire have a good reliability (Cronbach's alpha 0.93) and are scored according to a 5-point scale from disagreement to agreement (0—does not apply at all; 1—does not truly apply; 2—don't know (neither yes nor no); 3—applies quite a bit; 4—applies very much).^33^ The instrument does not use religious terminology and is, therefore, ideal for evaluating a patient's interest in spiritual/religious concerns without leaning toward or against a particular religious commitment.^34^

### Statistical analyses

Descriptive statistics and frequencies were used to characterize the participants of this study. Data are presented as mean and standard deviation (SD) or relative proportions (%).

Gender differences were assessed using independent *t*-tests and chi-square tests. The HADS cutoff score of patients >7 showed moderate symptoms and that of patients >11 showed severe symptoms of anxiety/depression.^32^ All reported *p*-values are two sided and considered significant when *p* < 0.05. Statistical analyses were performed with SPSS 26.0.

## Results

### Patient characteristics

Of the 689 participants, 207 (57.3%) women and 145 men (44.7%) reported being religious and/or spiritual. For our research questions, we only focused on the 352 (51.1%) patients with an R/S and excluded those without an R/S.

Of the 352 cancer patients with an R/S, a total of 350 filled out the HADS and 352 filled the Benefit and the socioeconomic characteristics. Missing items were not included in the analysis.

The participation in this survey was optional, and patients did not receive any benefits or experience disadvantages in their clinical treatment. The most common reasons for nonparticipation were health-related reasons such as fatigue or nausea.

The characteristics of the participants are given in Table 1. The mean age of the participants was 56.8, SD = 13.3 years (range between 18 and 89), 58.8% (207) were women with a mean age of 55.9 (SD = 12.35 years), and 41.2% were men (145) with a mean age of 58.0 (SD = 14.3 years). The majority of the R/S patients regarded themselves as religious (44.9% women, 49.7% men). More women reported being spiritual but not religious (19.8% vs. 14.5%). And 35.3% of women and 35.9% of men regarded themselves as religious and spiritual. There were no significant differences concerning the socioeconomic characteristics between men and women in this study, except the income. Concerning family status, more men than women in this study were married (65.5% vs. 55.6%), whereas more women were widowed (10.6% vs. 2.8%). More men had university degrees (25.0% vs. 16.4%) and were self-employed (11.0% vs. 5.8%), whereas women often had completed only compulsory levels of school and were more often homemakers (8.2% vs. 3.4%). This might also influence the income of the participants. Overall, men in this study had significantly more often a monthly income of >€2200 (34.5% vs. 22.2%), whereas women were more likely to have a monthly income <€1300 (26.1% vs. 17.9%).

**Table 1.** Differences of Demographic Characteristics by Gender

	Male 145 (41.2%)	Female 207 (58.8%)	*p*
Variable	N (%)	N (%)	
Age (mean, SD)	58.09 ± 14.28	55.95 ± 12.53	0.140
Age, years			
<31	6 (4.1)	6 (2.9)	
31–50	36 (24.8)	61 (29.5)	0.680
51–70	77 (53.1)	109 (52.7)	
>70	26 (17.9)	31 (15.0)	
Spiritual attitude			
Religious	72 (49.7)	93 (44.9)	
Spiritual	21 (14.5)	41 (19.8)	0.409
Religious and spiritual	52 (35.9)	73 (35.3)	
Marital status			
Married/cohabiting	104 (71.7)	129 (62.4)	
Divorced	23 (15.9)	33 (15.9)	0.09
Widowed	4 (2.8)	22 (10.6)	
Single	14 (9.7)	22 (10.6)	
Educational level			
Mandatory school	14 (9.7)	34 (16.4)	
Apprenticeship	67 (46.5)	89 (43.0)	0.11
High school graduation	25 (17.4)	45 (21.7)	
University	36 (25.0)	34 (16.4)	
Occupational status			
Self-employed	16 (11.0)	12 (5.8)	
Employee/laborer	41 (28.3)	77 (37.2)	0.08
Pension	69 (47.6)	87 (42.0)	
Homemaker/unemployed	17 (11.7)	26 (12.5)	
Student	1 (0.7)	1 (0.5)	
Income^a^			
<800	1 (0.7)	7 (3.4)	
800–1300	25 (17.2)	47 (22.7)	0.039^b^
1300–2200	48 (33.1)	66 (31.9)	
>2200	50 (34.5)	46 (22.2)	

*N* = number of participants.

^a^ Monthly income in Euro.

^b^ Significant differences between women and men (*p* < 0.05).

SD, standard deviation.

### The spiritual aspects of the participants

Table 2 displays the spiritual attitudes of the participants. Women scored higher than men in all items. The majority of the patients agreed that R/S plays a role in their life and cancer experience. In addition, 68.9% (142) of the women and 53.5% (76) of the men reported that their R/S promotes their inner strength in everyday life, and for 71% (144) of the women and 58.8% (83) of the men, R/S helps to have a deeper connection with their neighbors and the world around them. Also, 67.3% (138) of the women and 60.6% (86) of the men reported dealing with life more consciously because of their R/S. Regarding our research question of whether R/S plays a role in the coping process of a cancer journey, 75.1% (154) of women and 64.8% (92) of men mentioned that their R/S helped them to cope better with their illness. And 73.8% (152) of women and 60.4% (87) of men experience contentment and inner peace during R/S practices and 61% (125) of the women and 48.9% (69) of the men suggested that being engaged in R/S helps them to restore their mental and physical health.

**Table 2.** The Benefit Questionnaire Comparison Toward Religiosity and/or Spirituality Attitudes by Gender (Mean Values and Standard Deviation)

	Women	Men	*p*
	Mean ± SD	Mean ± SD	
5.1 When I practice my spirituality/religiosity, I generally experience contentment and inner peace.	2.99 ± 1.10	2.60 ± 1.22	0.002^a^
5.2 In everyday life, my spirituality or religiosity promotes my inner strength.	2.88 ± 1.14	2.42 ± 1.26	0.001^a^
4.2 My spirituality/religiosity brings a deeper connection with my neighbors and the world around me.	2.90 ± 1.11	2.62 ± 1.18	0.030^a^
4.3 My spirituality/religiosity helps me to manage my life more consciously	2.85 ± 1.09	2.63 ± 1.19	0.076
4.4 My spirituality/religiosity helps me to cope better with my illness.	2.96 ± 1.10	2.65 ± 1.25	0.017^a^
4.7 Being engaged in religion or spirituality helps to restore me to mental and physical health.	2.75 ± 1.16	2.48 ± 1.20	0.038^a^

^a^ Significant differences between women and men (*p* < 0.05).

### Psychological aspects

When examining anxiety and depression levels, significant gender differences were found.

As given in Table 3, women reported higher scores of anxiety (*M* = 6.39, SD = ±4.42) than men (*M* = 4.68, SD = ±3.83). The results of the *t*-test analysis revealed that these differences reached significance [*t*(348) = −3.77, *p* < 0.001].

**Table 3.** Comparison of Anxiety/Depression Levels by Gender

	Men	Women	*p*
	Mean ± SD	Mean ± SD	
Anxiety	4.68 ± 3.83	6.39 ± 4.42	<0.001^a^
Depression	4.42 ± 3.99	5.13 ± 4.04	0.109

^a^ Significant differences between women and men (*p* < 0.05).

According to the gender differences of the given HADS classification scores, women reported mild symptoms of anxiety (scores between 0 and 7) two times more often than men (21.4% vs. 9.7%). Severe levels of anxiety (scores >11) were reported by 17% of women and 10.4% of men.

Similar results were found for depression. The mean score for depression was higher in women (*M* = 5.13; SD ±4.04) than in men (*M* = 4.42, SD = ±3.99) [*t*(348) = −1.60, *p* = 0.109]. However, although both gender groups showed similar rates of mild symptoms of depression (13.1% vs. 12.7%), high levels of depression (scores >11) were found in 12.6% of women and 7.7% of men.

## Discussion

### Religious coping in cancer

The aim of our study was to explore gender differences in religious coping. To that end, we document differences in R/S in men and women.

Our first finding is that the majority of men (65%) and women (75%) confirmed that their R/S helps them to cope better with their cancer.

Dealing with a life-threatening disease inevitably brings feelings of powerlessness, anxiety, and uncertainty. Relying on R/S can give sources of comfort, strength, and hope in existential crises. A large body of literature highlights the importance of R/S in coping with cancer.^1–9^ In a recent mixed-methods study, Merath et al.^35^ also found that most of the cancer patients with an R/S belief affirmed that their belief played an important role in their cancer journey. Mesquita et al.^36^ observed in 101 patients undergoing chemotherapy treatment that religious/spiritual coping is a frequently used strategy among cancer patients and an important contribution to cope with the illness.

These results are in accordance with other studies, like one from Delgado-Guay et al.^7^ who interviewed 100 advanced cancer patients. Ninety-eight percent of the patients labeled themselves as religious and spiritual and confirmed that R/S beliefs strongly supported their coping process by providing strength and comfort.

### Women are more religious/spiritual

Women in this study were significantly more often religious/spiritual than men. This is in line with prior studies that showed similar results. Studies documented that women are more into religiousness and religious/spiritual practices and more frequently use R/S coping strategies than men. Büssing et al.^37^ found in chronically ill patients that women regard themselves as spiritual more than men and that spirituality and/or religion helps them to cope with their disease. Higher levels of religiosity were also found in female cancer survivors in an American cross-sectional study with ∼9000 cancer patients.^14^ Numerous studies have supported the idea of female religiosity. Klein^38^ mentioned “the pious women and the agnostic men.” Theories to explain these differences focused on biological, social, or general personality differences between women and men.^38,39^ Interestingly, Schnabel et al.^40^ point out that the majority of studies reporting on the higher frequency of women in religiosity and religious/spiritual practices are conducted in Christian populations. In contrast, this gender difference reverses when considering different religions. For example, among Muslims and Orthodox Jews, men participate more frequently in religion and religious practices than women. Schnabel et al.^40^ argue that “while one could say that Christian churches are a place for women, one could likewise say that Jewish synagogues are a place for men.” This, of course, also depends on the different socially constructed gender roles and tasks within Islam and Judaism. It can be said that gender gaps partially come from the differences in gendered norms, incentives, and expectations expressed in various religions.^40^ Hackett et al.^41^ examined the gender differences of religiosity and found that around the world, 83.4% of women and 79.9% of men identify with a faith group. They also concluded that women were more into religious practices worldwide.

The gender differences in R/S attitudes in this study were notable in patients who regarded themselves as spiritual, with more women than men considered to be spiritual but not religious. Steinhorn et al.^42^ suggest that people tend to shy away from institutional religion in secular societies. In contrast, more men than women in this study regarded themselves as religious but not spiritual. Feminist researchers argue that the trend of women moving away from traditional religions is based on gender inequalities^43^ and the patriarchal and misogynistic scriptures and practices they uphold.^44^

### Women scored higher on anxiety and depression

Another research question of this study asked whether there are differences between men and women concerning anxiety and depression and detected the influence of R/S.

Gender differences were found in the prevalence of cancer-related psychological symptoms. Women in this study experienced anxiety significantly more and scored higher on depression than men. Our findings are consistent with results from studies of patients with advanced cancer. Linden et al.^45^ described that female cancer patients in their study experienced feelings of anxiety almost two times more often than men (24.0% vs. 12.9%). Likewise, Ferrari^20^ found significantly lower scores for anxiety and depression in male cancer patients than in female patients. Numerous studies documented that female patients suffered more from anxiety and depression during their cancer experience and had a poorer quality of life than men.^46–49^ In addition, Martínez et al.^50^ reported that 25% of breast cancer patients reported clinical distress.

There is also an expanding literature supporting that R/S can decrease incidents of anxiety and depression in cancer patients and improve their quality of life.^51–53^

But why do women in this study suffer more with anxiety and depression symptoms during their cancer experience even though they are more religious/spiritual? This gender gap may rely more on gender-socialization effects than on gender differences in R/S. It can be argued that women more easily talk about their emotions and are more used to sharing their feelings and worries with others, whereas men tend to avoid disclosure of their disease and talking about their emotions; they prefer to manage things by themselves.^22,54^ Studies revealed differences in reporting and experiencing symptoms between men and women with cancer.^55^ Nozawa et al.^56^ found that women experienced greater distress and subsequently suffered more from those negative feelings than men. Similarly, Bergerot et al.^19^ discovered that there was a higher prevalence of reporting distress, anxiety, and depression among female patients than among male patients. Likewise, Lauriola and Tomai^57^ found that females experienced cancer with a greater degree of anxiety, depression, and feelings of helplessness than males. Women with cancer also tend to worry more about their condition than men, which leads to physical and psychological deterioration. It is difficult to determine whether the results depend on the differences between the reporting style of men and women or their actual experience of suffering.^46^ In contrast, Miller et al.^16^ investigated the risk of developing depression when facing stressful life circumstances. They found no significant difference in the prevalence between men and women.

### Gender role stereotypes

The literature presents evidence describing that men might show different signs of depression.^58^ Martin et al.^59^ identified typical male symptoms such as anger, aggression, substance abuse, and risky behavior. Price et al.^60^ state that the aforementioned male symptoms of depression do not necessarily occur naturally in higher levels in males but rather that these symptoms are indicative of depression in men. The ideals of men being strong, competitive, and avoiding showing emotion can be harmful to their physical and mental health.^61^ The differences between men and women can be explained according to gender role stereotypes,^62–64^ which greatly influence the experience with and sometimes the triggering of depression.^65^ Hegemonic masculinity can make it more difficult for men to seek help and employ adaptive coping.^66^ Gray et al.^22^ suggest that “men struggled to [stay in] control of their emotions and their lives, typically vacillating between the pulls of fierce self-reliance and fearful neediness.” This might be the reason why research documented that men with cancer have unmet needs.^67^ The ability to seek help still seems to be related to female competencies. Merckaert et al.^68^ reported that 25% of female cancer patients, as opposed to 10% of male cancer patients, expressed a need for psychological support. Psychological distress seems to be very differently recognized: although it is seen as a normal reaction for women, it is far too often unrecognized in men.^55^ To reduce this gender gap, “male-specific” assessments are used to detect depression in male cancer patients.^69^ Additional research is required for better understanding and treatment. Gender socialization can also influence the coping strategies of men and women while dealing with illness.^70^

Another potential reason for why we found higher scores of anxiety and depression in women in this study involves socioeconomic factors. Women were more likely to go through the treatment process alone due to being widowed and had lower income. Both factors are known to increase anxiety and depression symptoms.

Concerning the religious aspects, one explanation for higher anxiety and depression levels can be related to the fact that women feel more secure to express their feelings of sorrow and anxiety because of their R/S. The trust and belief in an omnipotent God or transcendence may give the feeling of being connected with something larger than oneself, which provides care, love, and security. Schreiber^71^ described a significant association between experiencing an intense engagement with God and less stress, anxiety, and depression in many breast cancer patients. Flannelly and Galek^72^ explain how and why the attachment theory is often used when explaining gender differences in religiosity. The basic notion in attachment theory is the importance of the secure base for a child to develop a secure attachment to its primary caregivers. For an adult believer, God can provide a secure base, and studies show that having a secure attachment to God is related to psychological well-being. In line with this, Jung^73^ found that the secure attachment to God only benefits women concerning mental health problems and general anxiety. Stokes^74^ argues that “women are more likely to describe religion as a ‘relationship with God,’ while men more often deem it a ‘set of beliefs.’”

Another aspect of gender differences in R/S is found in the R/S practices, church attendance, and using R/S as a coping mechanism.^75,76^ It is proven that religious and spiritual activities such as prayer groups, yoga, meditation, reiki, and secular meditation programs such as mindful-based stress reduction can improve feelings of comfort and peace and decrease psychosocial symptoms.^77–79^ Regarding gender differences in R/S practices, our study confirms that women find contentment and inner peace more than men. These findings are consistent with other research on R/S practices and gender that demonstrate higher religious/spiritual involvement and practices in women.^31,80,81^ Similarly, Jacobs-Lawson et al.^82^ found in lung cancer patients that women were more into spiritual practices and religious coping and showed fewer depressive symptoms than men. Religious/spiritual practices such as prayer, meditation, yoga, and others also contribute to a closer connection to one's own feelings and the inner self. This awareness of oneself and one's inner life makes it hard to suppress worries and sorrows, anxiety, anger, and all the feelings that come up while dealing with cancer. This may explain why women in this study reported more anxiety and depressive symptoms. At the same time, these practices can also help patients to feel secure and grounded when facing their emotions.

Results of previous research^83–85^ show a significant gender gap concerning the importance of R/S for patients and the psychological aspects of cancer. Both cancer and the coping strategies for handling this possibly life-threatening disease are complex, difficult, and challenging, even more so when we add the concepts of R/S alongside gender differences. This article has documented important factors concerning gender differences in anxiety and depression levels in cancer patients when implementing R/S as a possible resource for both men and women. It is important to see and understand these gender differences; it is just as important not to take on a simplistic view, which would reduce our ability to see and respect the individual experience of patients.^56^ There is a need for spotting both gender-specific and individual concerns in adapting psychosocial interventions for cancer patients.

Several limitations of this study should be noted. First, our study was conducted as a cross-sectional study; hence, our results do not claim causality. The majority of the participants lived in the capital of Austria, Vienna. We suppose that the R/S levels and the gender differences might differ outside of Vienna or in smaller hospitals. Therefore, we assume that the results cannot be generalized to Austrian cancer patients. Thus, further studies are required to verify our findings. Another limitation is that this study did not include qualitative questions that could demonstrate gender differences in more detail. A mixed-methods design would provide a better understanding of gender differences in the religious coping process to secure person-centered care.

## Conclusions

This study underscores the relevance of R/S themes in cancer patients and highlights an important gender difference. Women were more religious/spiritual and tended more to include their beliefs in the coping process. Our results also support the notion that cancer patients suffer from psychological distress and that women show more anxiety and depression symptoms. R/S may act as a buffer against psychological distress for both men and women.

The majority of the patients in this study confirmed that R/S resources helped them to cope with their illness. Thus, more research is needed to explore the meaning of R/S. To provide holistic patient-centered care, the religious/spiritual and gender aspects should be included in the daily routine work. The gender perspective is essential and can contribute to better patient care in identifying gender-specific concerns. Further studies are needed to understand the gender differences in coping with cancer so that anxiety and depression can be recognized and treated with regard to the specific needs and struggles of men and women with cancer.

## Abbreviations Used

HADS: Hospital Anxiety and Depression Scale
R/S: religiosity and/or spirituality
SD: standard deviation
